# The significance of anti-neuronal antibodies for acute psychiatric disorders: a retrospective case–controlled study

**DOI:** 10.1186/s12868-018-0471-7

**Published:** 2018-11-03

**Authors:** Morten B. Schou, Sverre Georg Sæther, Ole Kristian Drange, Karoline Krane-Gartiser, Solveig K. Reitan, Arne E. Vaaler, Daniel Kondziella

**Affiliations:** 1 Department of Mental Health, Norwegian University of Science and Technology (NTNU), Trondheim, Norway; 2 Division of Mental Health Care, St Olavs Hospital HF, avd Østmarka, Trondheim University Hospital, Postboks 3250, Torgarden, 7006 Trondheim, Norway; 3 Division of Mental Health Care, St Olavs Hospital HF, Nidaros DPS, Trondheim University Hospital, Postboks 3250, Torgarden, 7006 Trondheim, Norway; 4 Division of Mental Health Care, Tiller DPS, St Olavs Hospital HF, Trondheim University Hospital, Postboks 3250, Torgarden, 7006 Trondheim, Norway; 5 Neurology Department, Rigshospitalet, Copenhagen University Hospital, Blegdamsvei 9, 2100 København Ø, Denmark

**Keywords:** Mental disorders, Psychoneuroimmunology, Anti-neuronal antibodies, NMDA receptor antibodies

## Abstract

**Background:**

The clinical significance of anti-neuronal antibodies in patients with psychiatric disorders, but without encephalitis, remains unknown. In patients admitted to acute psychiatric inpatient care we aimed to identify clinical features distinguishing anti-neuronal antibody positive patients from matched controls.

**Results:**

Patients who were serum-positive to *N*-methyl d-aspartate receptor (NMDAR) (n = 21), contactin-associated protein 2 (CASPR2) (n = 14) and/or glutamic acid decarboxylase 65 (GAD65) (n = 9) antibodies (cases) were age and sex matched (1:2) with serum-negative patients from the same cohort (controls). The prevalence and severity of psychiatric symptoms frequently encountered in NMDAR, CASPR2 and GAD65 antibody associated disorders were compared in cases and controls. NMDAR, CASPR2 and GAD65 antibody positive patients did not differ in their clinical presentation from matched serum negative controls.

**Conclusion:**

In this cohort, patients with and without NMDAR, CASPR2 and GAD65 antibodies admitted to acute psychiatric inpatient care had similar psychiatric phenotypes. This does not exclude their clinical relevance in subgroups of patients, and studies further investigating the clinical significance of anti-neuronal antibodies in patients with psychiatric symptomatology are needed.

**Electronic supplementary material:**

The online version of this article (10.1186/s12868-018-0471-7) contains supplementary material, which is available to authorized users.

## Background

Anti-neuronal antibodies are associated with autoimmune encephalitis, which often presents with psychiatric symptoms [1]. We recently found serum anti-neuronal antibodies [Immunoglobulin (Ig) G, IgA and/or IgM] in 12% of 925 patients consecutively admitted to acute psychiatric inpatient care [*N*-methyl d-aspartate receptor (NMDAR) antibodies in 7.6%, contactin-associated protein 2 (CASPR2) antibodies in 2.5%, and glutamic acid decarboxylase 65 (GAD65) antibodies in 1.9%] [2]. The IgG isotype of NMDAR, CASPR2 and GAD65 antibodies has been associated with autoimmune encephalitis with prominent psychiatric features [1]. The IgA and IgM isotypes of NMDAR antibodies have been associated with psychotic symptoms in dementia [3, 4], and there is some evidence that they have pathogenic potential [5]. In a recent meta-analysis, Grain et al. found that GAD65 antibodies are more prevalent in patients with psychotic disorders compared to controls [6]. The role of any of these antibodies in psychiatric patients without evidence of autoimmune encephalitis is, however, not clear. This is an important issue to address because these patients might benefit from immunotherapy [7].

The prevalence of anti-neuronal antibodies in patients with psychiatric disorders has been investigated in several studies [2, 8–10]. However, it might be that the traditional psychiatric diagnostic classifications [e.g. International Classification of Diseases-10 (ICD-10)] are inadequate for the plethora of autoimmune psychiatric symptoms [11, 12]. Consequently, we chose a different approach. In this large single-center study, we searched for differences in the clinical phenotypes of patients admitted to acute psychiatric inpatient care who tested either positive or negative for three well-known anti-neuronal antibodies (NMDAR, CASPR2 and GAD65). We hypothesized that psychiatric patients testing positive to a specific antibody (e.g. anti-NMDAR) would have an increased frequency and/or severity of psychiatric symptoms typically seen in neurological syndromes associated with that antibody (e.g. anti-NMDAR encephalitis).

## Methods

### Setting

This case-controlled study was performed in an acute psychiatric inpatient clinic in a university center (St. Olavs Hospital, Trondheim University Hospital, Trondheim, Norway). The hospital receives all patients (≥ 18 years) admitted to acute psychiatric inpatient care in the catchment area. The most common reasons for referral include major depression, bipolar disorder, schizophrenia spectrum disorders, personality disorders, anxiety disorders or substance induced psychiatric disorders. The only inclusion criterion was admission to acute psychiatric inpatient care. Exclusion criteria were inability to give informed consent, discharge before consent could be obtained, or lack of proficiency in Norwegian or English.

### Patients

**Fig. 1:**
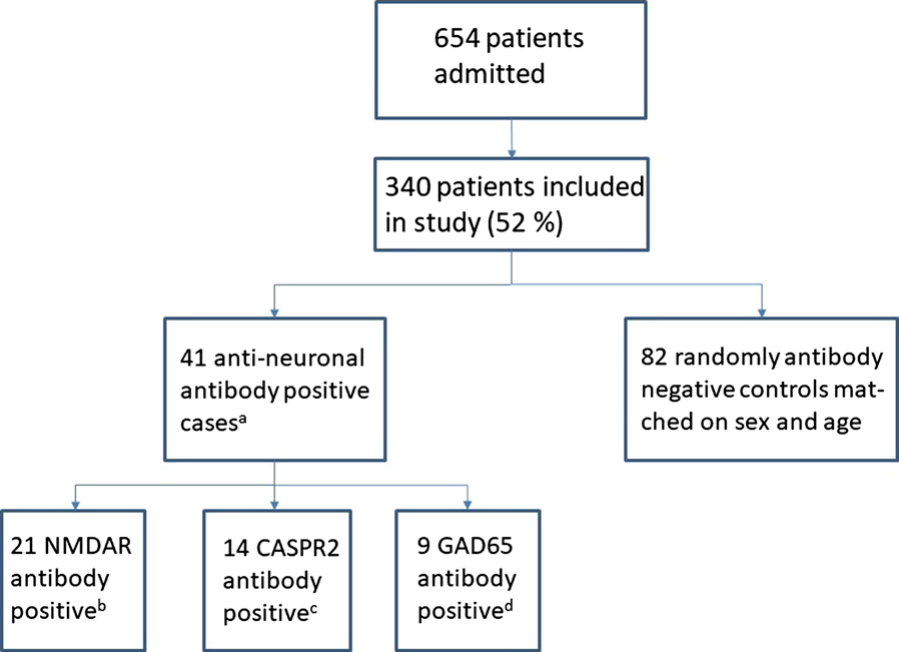
Flow chart over patient recruitment, cases, and controls. ^a^Three patients were positive for both NMDAR and GAD65 antibodies. ^b^Immunglobulin (Ig) isotype 11/3/9 (IgA/IgG/IgM), titer, median (range) 1:32 (1:10–1:1000), 2 patients were positive to both NMDAR IgM and IgA antibodies. ^c^Ig isotype 2/6/6 (IgA/IgG/IgM) titer, median (range) 1:10 (1:10–1:100). ^d^ Ig isotype 1/8/0 (IgA/IgG/IgM), titer, median (range) 1:10 (1:10–1:320). See Additional file 1 for full list of antibody isotype and endpoint titer. *CASPR2* contactin-associated protein 2, *GAD65* glutamic acid decarboxylase 65, *Ig* immunoglobulin, *NMDAR N*-methyl-d-aspartate receptor

A total of 654 consecutive patients were admitted during 7 months in 2011–2012. Three hundred and forty patients (52%) consented to participate in the study, of which 41 tested positive for NMDAR, CASPR2 and/or GAD65 antibodies (IgA, IgG or IgM). None tested positive for antibodies directed to Leucine-rich glioma-inactivated protein 1 (LGI1), α-amino-3-hydroxy-5-methyl-4-isoxazolepropionic acid receptor (AMPAR) or γ-aminobutyric acid B receptor (GABA_B_R) [2]. Eighty-two anti-neuronal antibody negative controls were chosen from the same cohort (i.e. 2 controls for each case) (Fig. 1). Controls were selected randomly among patients with the same sex and age (± 5 years) as each case. If no such patient was present in the cohort, the age interval was increased (± 10 years, ± 15 years).

### Variables

Variables of symptomatology were selected following a systematic literature search for psychiatric symptomatology in disorders associated with NMDAR, CASPR2 and GAD65 antibodies. Comparisons were made only for symptoms associated with each specific anti-neuronal antibody. See Additional file 1 for search strategy and citations on the papers reviewed. Symptom variables were included only if they either were available from the data collected during the inclusion period (2011–2012) or could be reliably assessed during retrospective chart review. Symptom variables included were; hallucinations, delusions, lowered mood, elevated mood, irritability, disinhibition, agitation, disorientation, symptom fluctuation, and sleep problems. The symptom variables anxiety, catatonia and apathy were also extracted in the literature review but were deemed too unreliable to be assessed by retrospective chart review. A subset of symptom variables was associated with exclusively one or two of the antibodies assessed in this study (Table 1).

**Table 1:** Patients with and without anti-neuronal antibodies were compared on the following clinical characteristics

Clinical characteristic	NMDAR	CASPR2	GAD65	Variable	Obtained	Definitions
Hallucinations	X	X	n/a	Dichotome (Yes/No)	Retrospective	Vis., aud., tact. and/or olf.
Delusions	X	X	n/a	Dichotome (Yes/No)	Retrospective	Described in chart
Lowered mood	X	X	X	Dichotome (Yes/No)	Retrospective	Described in chart
Elevated mood	X	n/a	n/a	Dichotome (Yes/No)	Retrospective	Described in chart
Irritability	X	n/a	X	Dichotome (Yes/No)	Prospective	Brøset Violence Checklist
Disorientation	X	X	X	Dichotome (Yes/No)	Prospective	Brøset Violence Checklist
Disinhibition	X	n/a	n/a	Continuous (1–7)	Prospective	PANSS Item G14
Agitation	X	X	X	Continuous (5–35)	Prospective	PANSS-EC
Symptom fluctuation	X	n/a	n/a	Continuous (1–10)	Prospective	SOMAS Item A
Total sleep time (actigraphy)	X	X	n/a	Continuous (min)	Prospective	According to actigraphy software algorithms
Wake after sleep onset (actigraphy)	X	X	n/a	Continuous (min)	Prospective	According to actigraphy software algorithms

*aud* auditive, *CASPR2* contactin-associated protein 2, *GAD65* glutamic acid decarboxylase 65, *n/a* not applicable (because the systematic literature search did not reveal associations between the symptom variable and the specific antibody), *NMDAR N*-methyl-d-aspartate receptor, *olf* olfactory, *PANSS* positive and negative syndrome scale, *PANSS-EC* positive and negative syndrome scale- excited component, *SOMAS* Symptomatic Organic Mental Disorder Assessment Scale, *tact* tactile, *Vis* visual

On the first day following admission, the attending physicians evaluated the degree of agitation with the Positive and Negative Syndrome Scale-Excited Component (PANSS-EC) [13], impulse control as a measure of disinhibition with the use of PANSS item G14 and the degree of fluctuation of psychiatric symptoms with Symptomatic Organic Mental Disorder Assessment Scale (SOMAS) item A [14]. In addition, the nursing staff evaluated the degree of irritability and disorientation with the Brøset Violence checklist (BVC) [15]. Sleep variables were recorded by an actigraph worn around the wrist for 24 h soon after admission (Actiwatch Spectrum, Philips Respironics Inc., Murrysville PA, USA) [16], mean time until the actigraphy recording started was 2.2 (SD 2.2) days after admission. A blinded assessor scored the actigraphy recordings. For each patient a rest interval at nighttime was set by visual inspection. The actigraphy software (Actiware, version 5.70.1) then automatically calculated the variables “total sleep time” and “wake after sleep onset” during the rest interval using the Immobile Minutes algorithm of 10 min, and a wake threshold after sleep onset of 40 activity counts (medium sensitivity), which has been used in validation studies [17, 18]. All other clinical characteristics were extracted from patient charts by blinded examiners who reviewed charts from the 24 h following admission. Psychiatric diagnoses were set according to the International Classification of Diseases (ICD)-10 criteria for research [19] in a consensus meeting including the physician or psychologist in charge of the treatment of the patient and at least two psychiatrists and/or senior clinical psychologist. The main diagnosis was registered in this study. Patients were asked for life-time history of seizures and evaluated with regards to whether or not alcohol or illegal substances had been consumed during the days/weeks prior to admission. This evaluation consisted of patient interviews, alcohol breathing tests and urine analyses of alcohol, benzodiazepines (oxazepam, desmethyldiazepam, nitrazepam, flunitrazepam, clonazepam, and alprazolam), zopiclone, stimulants (amphetamine, metamphetamine, 3,4-methylendioksymetamphetamine, 3,4-methyl-dioxy-amphetamine, ephedrine, and benzoylecgonine), opioids (morphine, codeine, etylmorphine, methadone, buprenorphine, pholcodine, and oxycodone) carisoprodol, meprobamate, cannabis, and phencyclidine (Liquid chromatography with mass spectroscopy).

### Serological analysis

Sera were tested for the presence of anti-neuronal antibodies directed against NMDAR, LGI1, CASPR2, AMPAR, GABA_B_R and GAD65 (IgA, IgG and IgM) using transfected HEK293 cells expressing the respective recombinant target antigens (Euroimmun, Lübeck, Germany) [20, 21]. Samples were classified as positive or negative based on fluorescence intensity of the transfected cells in direct comparison with non-transfected cells and control samples. Endpoint titers were defined as the last dilution showing a measurable degree of fluorescence, with 1:10 being the cut-off for positivity [20, 21].

### Ethics

On the day after admission a psychiatrist or senior clinical psychologist evaluated each patient’s ability to consent. Patients without ability to consent were excluded. Included patients gave written, informed consent. The study was conducted in accordance with the Declaration of Helsinki and approved by The Regional Committee for Medical Research Ethics, Central Norway (2011/137). The data for the present study were collected as part of a previous clinical trial, “Agitation in the Acute Psychiatric Department”, which was prospectively registered on https://clinicaltrials.gov/ on August 11th 2011 (NCT01415323).

### Statistics

We compared patients with a positive serology for NMDAR, CASPR2 or GAD65 antibodies with their respective age- and sex-matched controls for the presence and degree of psychiatric symptoms as outlined in Tables 1 and 3. Categorical variables were analyzed using the Chi square test or Fisher’s exact test. Continuous variables were compared using the *T* test or Mann–Whitney U-test. Alpha level was set at 0.05. Adjustment for multiple comparisons was not performed due to the exploratory study design. Statistical analyses were done in SPSS 21 (SPSS, Chicago, US-IL).

## Results

### Demographic and clinical data

The inclusion rate was 52% (340 out of 654 admitted patients). There were no significant differences in age (p = 0.64, Mann–Whitney U test) or sex (p = 0.67, chi- square test) between included and non-included patients. However, there was a difference in diagnostic distribution between the groups (p < 0.001, Chi square test). This was attributable to overrepresentation of patients suffering from depressive and bipolar disorders, and underrepresentation of patients suffering from psychotic disorders and patients not fulfilling ICD-10 criteria for a specific psychiatric disorder (Z-diagnosis) among the included patients (data not shown).

Demographic and clinical data of cases and controls are presented in Table 2. Compared to controls, NMDAR antibody positive patients had a higher prevalence of alcohol and substance use prior to admission (76 vs. 50%, p = 0.047) and received antidepressant drugs more often at discharge (43 vs. 17%, p = 0.024). GAD65 antibody positive patients received higher doses of antipsychotic medication compared to controls both at admission and discharge [Chlorpromazine equivalents mean (SD) 1071 (124) vs. 469 (276), p = 0.013, and 784 (403) versus 331 (254), p-value = 0.015, respectively]. None of the anti-GAD65 positive cases or controls had diabetes mellitus type I.

**Table 2:** Demographic and clinical data of patients with NMDAR, CASPR2 or GAD65 antibodies and of their controls

	NMDAR		CASPR2		GAD65	
	Cases (n = 21)	Controls (n = 42)	Cases (n = 14)	Controls (n = 28)	Cases (n = 9)	Controls (n = 18)
Age, mean (SD)	48.6 (16.3)	46.7 (14.2)	45.0 (16.1)	43.4 (14.7)	47.1 (14.0)	45.8 (11.8)
Sex, men (%)	62	62	71	71	56	56
Education, n (%)						
≤ 9 years	9 (43)	20 (48)	6 (43)	9 (32)	4 (44)	7 (39)
10–12 years	7 (33)	14 (33)	2 (14)	13 (46)	4 (44)	9 (50)
> 12 years	5 (24)	8 (19)	6 (43)	6 (21)	1 (11)	2 (11)
Psychiatric diagnosis, n (%)						
Substance use disorder (F10–19)	4 (19)	7 (17)	3 (21)	6 (21)	2 (22)	3 (17)
Psychotic disorder (F20–29)	1 (6)	6 (14)	2 (14)	6 (21)	1 (11)	1 (6)
Affective disorder (F30–39)	8 (38)	20 (48)	5 (36)	12 (43)	3 (33)	9 (50)
Other psychiatric disorders^a^	8 (38)	9 (21)	4 (29)	4 (14)	3 (33)	5 (28)
Psychopharmacological med. at admission, n (%)						
Antipsychotic med.	6 (29)	13 (31)	5 (36)	10 (36)	3 (33)	5 (28)
Antipsychotic dose, mean (SD)^b^	482 (422)	458 (302)	241 (185)	324 (170)	1071 (124)	469 (276)*
Antidepressive med.	8 (38)	9 (21)	4 (29)	8 (29)	3 (33)	6 (33)
Mood stabilizing med.	3 (14)	10 (24)	2 (14)	4 (14)	2 (22)	3 (17)
No psychopharmacological med.	10 (48)	18 (43)	5 (36)	12 (43)	4 (44)	8 (44)
Psychopharmacological med. at discharge, n (%)						
Antipsychotic med.	9 (43)	26 (62)	6 (43)	18 (64)	5 (56)	11 (61)
Antipsychotic dose, mean (SD)^b^	418 (449)	408 (331)	222 (203)	342 (226)	784 (403)	331 (254)*
Antidepressive med.	9 (43)	7 (17)*	4 (29)	7 (25)	2 (22)	6 (33)
Mood stabilizing med.	6 (29)	15 (36)	5 (36)	6 (21)	2 (22)	5 (28)
No psychopharmacological med.	5 (24)	10 (24)	3 (21)	5 (18)	2 (22)	5 (28)
Number of days admitted, mean (SD)	9.5 (11.4)	9.9 (9.1)	9.5 (6.1)	10.1 (11.6)	9.6 (9.3)	9.1 (8.3)
Alcohol or substance use days/weeks prior to admission, n (%)	16 (76)	21 (50)*	10 (71)	18 (64)	7 (78)	10 (56)
History of seizures^c^	1 (6)	9 (25)	4 (40)	3 (13)	2 (22)	3 (18)

*CASPR2* contactin-associated protein 2, *eq* equivalents, *GAD65* glutamic acid decarboxylase 65, *med* medication, *NMDAR N*-methyl-d-aspartate receptor, *SD* standard deviation

*p < 0.05

^a^3 patients with organic mental disorder (F00–09), 13 patients with anxiety disorders (F40–49), 7 patients with personality disorders (F60–69), 1 patient with mental retardation (F70–79), 1 patient with ADHD (F90–98) and 5 patients without specific psychiatric disorder (Z00–99); ^b^chlorpromazine equivalents; ^c^self-reported at admission (missing data; NMDAR, 3 cases and 6 controls; CASPR2, 4 cases and 5 controls; GAD, 1 control

### Clinical characteristics

None of the clinical parameters differed between patients with NMDAR, CASPR2 and GAD65 antibodies and their respective controls (Table 3). None of the NMDAR IgG positive patients had symptoms or signs of NMDAR encephalitis.

**Table 3:** Psychiatric symptoms in antibody positive cases (+) and controls (−)

Clinical characteristic	NMDAR		p^a^	CASPR2		p^a^	GAD65		
	+ n = 21	− n = 42		+ n = 14	− n = 28		+ n = 9	− n = 18	p^a^
Hallucinations, n (%)	3 (14.3)	1 (2.4)	0.10	0 (0)	3 (10.7)	0.54			
Delusions, n (%)	2 (9.5)	7 (16.7)	0.71	2 (14.3)	3 (10.7)	1.00			
Lowered mood^b^, n (%)	10 (55.6)	16 (39.0)	0.24^i^	8 (61.5)	15 (60.0)	0.93^i^	5 (55.6)	11 (64.7)	0.69
Elevated mood^c^, n (%)	2 (11.1)	7 (17.1)	0.71						
Irritability^d^, n (%)	3 (15.0)	6 (14.3)	1.00	3 (23.1)	5 (18.5)	1.00	1 (12.5)	5 (27.8)	0.63
Disorientation^e^, n (%)	1 (5.0)	7 (16.7)	0.26	4 (30.8)	4 (15.4)	0.40	0 (0)	2 (11.1)	1.00
Disinhibition (median (range))	1 (1–6)	1 (1–6)	0.57^j^						
Agitation (median (range))^f^	8 (5–31)	8 (5–32)	0.62^j^	7 (5–27)	10 (5–21)	0.34^j^	10 (5–17)	8 (5–23)	0.98^j^
Symptom fluctuation (median (range))^g^	2 (1–7)	3 (1–8)	0.89^j^						
Total sleep time (min) (mean (SD))^h^	458 (115)	476 (112)	0.66^k^	438 (109)	442 (114)	0.93^k^			
Time awake after sleep onset (min) (mean (SD))^h^	39 (23)	37 (35)	0.90^k^	47 (18)	40 (24)	0.51^k^			

*CASPR2* contactin-associated protein 2, *GAD65* glutamic acid decarboxylase 65, *NMDAR N*-methyl-d-aspartate receptor, *SD* standard deviation

^a^Fisher’s exact test if not stated otherwise. Data missing on ^b^NMDAR (3 cases, 1 control), CASPR2 (1 case, 3 controls), GAD65 (1 control); ^c^NMDAR (3 cases, 1 control); ^d^NMDAR (1 case), CASPR2 (1 case, 1 control), GAD65 (1 case); ^e^NMDAR (1 case), CASPR2 (1 case, 2 controls), GAD65 (1 case); ^f^CASPR2 (1 case, 1 control); ^g^NMDAR (3 cases, 7 controls); ^h^NMDAR (10 cases, 16 controls), CASPR2 (6 cases, 14 controls); ^i^Chi square; ^j^Mann Whitney U test; ^k^T-test

## Discussion

In this large cohort of patients admitted to acute psychiatric inpatient care, patients who were serum positive or negative to anti-neuronal antibodies had a similar psychiatric phenotype. Specifically, patients with NMDAR, CASPR2 and GAD65 antibodies did *not* exhibit psychiatric symptoms suggestive of autoimmune encephalitis more frequently than controls.

Previous studies in patients with psychiatric disorders have explored the prevalence of anti-neuronal antibodies in different diagnostic groups. It is still controversial whether or not the prevalence of anti-neuronal antibodies is increased in patients with first episode or chronic psychosis [2, 8–10, 22, 23]. A limited number of studies have addressed clinical characteristics in anti-neuronal antibody-positive and -negative psychiatric patients irrespective of diagnostic categories. Hammer et al. [24] did not find any differences in PANSS or Global Assessment of Function (GAF) when comparing patients with schizophrenia who were positive or negative for NMDAR antibodies. Similarly, in a cohort of patients with first-episode psychosis PANSS scores, cognitive testing and catatonia symptoms were not clinically significant different in anti-neuronal antibody positive (NMDAR, CASPR2, LGI1 or GABA_A_ receptor antibodies) and negative patients [9]. The authors of a study including patients with both first episode and chronic schizophrenia found more severe psychotic symptoms (PANSS scores) in NMDAR antibody positive compared to negative patients [10]. The studies in this field are heterogeneous and the results depend to a certain degree on the antibody detection method used. Fixed and live cell-based assays are the most commonly used methods for anti-neuronal antibody detection. Using a novel single molecule-based imaging approach, Jezequel et al. [10] recently showed that NMDAR antibodies from schizophrenia patients alter the surface dynamics of the NMDAR in contrast to NMDAR antibodies from healthy controls. Jezequel et al. [25] found that fixed cell-based assays (such as the one used in this study) have a lower sensitivity for detection of IgG antibodies in psychotic patients compared to live cell-based assays. Hence, it is possible that the use of other antibody detection methods in the present study would have yielded slightly different results. Another possible explanation for the lack of phenotypic differences is the low antibody titers found in our patients; alternatively, the lack of significant findings in our study could reflect a lack of clinical significance of these antibodies for acute psychiatric patients in general. Hence, whether or not phenotypical differences are present in psychiatric patients with higher antibody titers is an important question for further research. To further investigate this, future studies should include cerebrospinal fluid (CSF) analyses, electroencephalography (EEG) and brain imaging.

NMDAR antibody positive patients were treated more often with antidepressants than controls. These findings could be coincidental. However, it is also possible that excessive use of antidepressants indicates a higher burden of depressive and/or anxious symptoms in NMDAR positive patients, although we were unable to detect such differences in our retrospective chart assessment. The increased frequency of alcohol and substance use prior to admission in NMDAR antibody positive patients may suggest self-medication for depressive and/or anxious symptoms. However, an influence of alcohol and substance use on NMDAR antibody titers cannot be ruled out. The NMDAR is implicated in addiction in several ways. For instance, associations have been found between addiction and genes coding for NMDAR subunits [26]; alcohol has acute and chronic effects on NMDAR functioning [27]; and NMDAR modulators are used to treat alcohol dependency [28]. Interestingly, alcohol and illicit substances can cause blood brain barrier dysfunction [29, 30], which might facilitate the occurrence of NMDAR antibodies by exposing NMDAR to lymphoid cells. However, the exact reasons for the observed association between NMDAR antibodies and alcohol and substance remains unknown. GAD65 antibody positive patients used higher doses of antipsychotic drugs compared to antibody negative patients, which could imply a more severe symptomatology in these patients. Alternatively, antipsychotic medication might also lead to enhanced production of GAD65 antibodies. A similar association is known for chlorpromazine and antinuclear antibodies [31, 32].

The present study has limitations. The inclusion rate of 52% is similar to other studies in this setting [33, 34]. However, there is a risk of selection bias (i.e. patients with a higher severity of symptoms may decline participation or lack ability to consent more often than patients with less severe phenotypes). Patients with affective disorders were overrepresented and psychotic disorders underrepresented in our study. We included patients with all isotypes of NMDAR, CASPR2 and GAD65 antibodies (IgG, IgA and IgM). Whereas most known relevant anti-neuronal antibodies are of the IgG isotype, the results of pathogenicity studies of NMDAR IgA and IgM antibodies show their pathogenic potential in vitro [5, 24, 35, 36] and in a study of patients with stroke [37], although authors from another study concluded that NMDAR IgA and IgM antibodies do not alter NMDAR levels [38]. It is possible that our study would have yielded a different result if we had focused exclusively on IgG positive patients. Also, small group sizes and the categorical nature of several of the variables may have resulted in a lower sensitivity for detecting clinical differences. Although age- and sex-matched control subjects were randomly selected, some differences in diagnostic distribution and psychopharmacological treatment between the case and control group were present (Table 3).

## Conclusion

Based on our findings, patients admitted to acute psychiatric care with and without NMDAR, CASPR2 and GAD65 antibodies have a similar clinical phenotype. However, of note, absence of phenotypic differences between patients with and without anti-neuronal antibodies is not evidence that these antibodies lack clinical significance. Even if anti-neuronal antibodies played a role in only a minor subset of psychiatric patients, this would have important clinical implications as these patients might benefit from immunomodulatory treatment. This area must be further investigated by large prospective longitudinal multicenter studies that include cerebrospinal fluid analyses, brain imaging and electrophysiological investigations.

## Additional file

**Additional file 1.**Search strategy for selection of variables: search strategy and citations reviewed during variable selection; Anti-neuronal antibody status in cases: antibody type, subtype and titer in all cases.

## Abbreviations

BVC: Brøset violence checklist
CASPR2: contactin-associated protein 2
GAD65: glutamic acid decarboxylase 65
ICD: international classification of diseases
Ig: immunoglobulin
NMDAR: *N*-methyl d-aspartate receptor
PANSS-EC: Positive and Negative Syndrome Scale-Excited Component
SOMAS: Symptomatic Organic Mental Disorder Assessment Scale

## References

[1] Herken J, Pruss H (2017). Red flags: clinical signs for identifying autoimmune encephalitis in psychiatric patients. Front Psychiatry/Front Res Found.

[2] Schou M, Saether SG, Borowski K, Teegen B, Kondziella D, Stoecker W (2016). Prevalence of serum anti-neuronal autoantibodies in patients admitted to acute psychiatric care. Psychol Med.

[3] Busse S, Busse M, Brix B, Probst C, Genz A, Bogerts B (2014). Seroprevalence of *N*-methyl-d-aspartate glutamate receptor (NMDA-R) autoantibodies in aging subjects without neuropsychiatric disorders and in dementia patients. Eur Arch Psychiatry Clin Neurosci.

[4] Busse S, Brix B, Kunschmann R, Bogerts B, Stoecker W, Busse M (2014). *N*-methyl-d-aspartate glutamate receptor (NMDA-R) antibodies in mild cognitive impairment and dementias. Neurosci Res.

[5] Castillo-Gomez E, Oliveira B, Tapken D, Bertrand S, Klein-Schmidt C, Pan H (2017). All naturally occurring autoantibodies against the NMDA receptor subunit NR1 have pathogenic potential irrespective of epitope and immunoglobulin class. Mol Psychiatry.

[6] Grain R, Lally J, Stubbs B, Malik S, LeMince A, Nicholson TR (2017). Autoantibodies against voltage-gated potassium channel and glutamic acid decarboxylase in psychosis: a systematic review, meta-analysis, and case series. Psychiatry Clin Neurosci.

[7] Zandi MS, Deakin JB, Morris K, Buckley C, Jacobson L, Scoriels L (2014). Immunotherapy for patients with acute psychosis and serum *N*-methyl d-Aspartate receptor (NMDAR) antibodies: a description of a treated case series. Schizophr Res.

[8] Dahm L, Ott C, Steiner J, Stepniak B, Teegen B, Saschenbrecker S (2014). Seroprevalence of autoantibodies against brain antigens in health and disease. Ann Neurol.

[9] Lennox BR, Palmer-Cooper EC, Pollak T, Hainsworth J, Marks J, Jacobson L (2017). Prevalence and clinical characteristics of serum neuronal cell surface antibodies in first-episode psychosis: a case–control study. Lancet Psychiatry.

[10] Jezequel J, Johansson EM, Dupuis JP, Rogemond V, Grea H, Kellermayer B (2017). Dynamic disorganization of synaptic NMDA receptors triggered by autoantibodies from psychotic patients. Nat Commun.

[11] Pollak TA, Beck K, Irani SR, Howes OD, David AS, McGuire PK (2016). Autoantibodies to central nervous system neuronal surface antigens: psychiatric symptoms and psychopharmacological implications. Psychopharmacology.

[12] Pearlman DM, Najjar S (2014). Meta-analysis of the association between *N*-methyl-d-aspartate receptor antibodies and schizophrenia, schizoaffective disorder, bipolar disorder, and major depressive disorder. Schizophr Res.

[13] Montoya A, Valladares A, Lizan L, San L, Escobar R, Paz S (2011). Validation of the Excited Component of the Positive and Negative Syndrome Scale (PANSS-EC) in a naturalistic sample of 278 patients with acute psychosis and agitation in a psychiatric emergency room. Health Qual Life Outcomes.

[14] Vaaler AE, Morken G, Iversen VC, Kondziella D, Linaker OM (2010). Acute Unstable Depressive Syndrome (AUDS) is associated more frequently with epilepsy than major depression. BMC Neurol.

[15] Woods P, Almvik R (2002). The Broset violence checklist (BVC). Acta Psychiatr Scand Suppl.

[16] Ancoli-Israel S, Cole R, Alessi C, Chambers M, Moorcroft W, Pollak CP (2003). The role of actigraphy in the study of sleep and circadian rhythms. Sleep.

[17] Paquet J, Kawinska A, Carrier J (2007). Wake detection capacity of actigraphy during sleep. Sleep.

[18] Kaplan KA, Talbot LS, Gruber J, Harvey AG (2012). Evaluating sleep in bipolar disorder: comparison between actigraphy, polysomnography, and sleep diary. Bipolar Disord.

[19] WHO (1993). The ICD-10 classification of mental and behavioural disorders: diagnostic criteria for research.

[20] Probst C, Saschenbrecker S, Stoecker W, Komorowski L (2014). Anti-neuronal autoantibodies: current diagnostic challenges. Mul Scler Relat Disord.

[21] Wandinger KP, Saschenbrecker S, Stoecker W, Dalmau J (2011). Anti-NMDA-receptor encephalitis: a severe, multistage, treatable disorder presenting with psychosis. J Neuroimmunol.

[22] Masdeu JC, Gonzalez-Pinto A, Matute C, Ruiz De Azua S, Palomino A, De Leon J (2012). Serum IgG antibodies against the NR1 subunit of the NMDA receptor not detected in schizophrenia. Am J Psychiatry.

[23] Pathmanandavel K, Starling J, Merheb V, Ramanathan S, Sinmaz N, Dale RC (2015). Antibodies to surface dopamine-2 receptor and *N*-methyl-d-aspartate receptor in the first episode of acute psychosis in children. Biol Psychiat.

[24] Hammer C, Stepniak B, Schneider A, Papiol S, Tantra M, Begemann M (2014). Neuropsychiatric disease relevance of circulating anti-NMDA receptor autoantibodies depends on blood-brain barrier integrity. Mol Psychiatry.

[25] Jezequel J, Rogemond V, Pollak T, Lepleux M, Jacobson L, Grea H (2017). Cell- and single molecule-based methods to detect anti-*N*-methyl-d-aspartate receptor autoantibodies in patients with first-episode psychosis from the OPTiMiSE Project. Biol Psychiat.

[26] Chen J, Ma Y, Fan R, Yang Z, Li MD (2018). Implication of genes for the *N*-methyl-d-aspartate (NMDA) receptor in substance addictions. Mol Neurobiol.

[27] Ron D, Wang J, Van Dongen AM (2009). The NMDA receptor and alcohol addiction. Biology of the NMDA receptor.

[28] Tomek SE, Lacrosse AL, Nemirovsky NE, Olive MF (2013). NMDA receptor modulators in the treatment of drug addiction. Pharmaceuticals (Basel).

[29] Kousik SM, Napier TC, Carvey PM (2012). The effects of psychostimulant drugs on blood brain barrier function and neuroinflammation. Front Pharmacol.

[30] Rubio-Araiz A, Porcu F, Perez-Hernandez M, Garcia-Gutierrez MS, Aracil-Fernandez MA, Gutierrez-Lopez MD (2017). Disruption of blood-brain barrier integrity in postmortem alcoholic brain: preclinical evidence of TLR4 involvement from a binge-like drinking model. Addict Biol.

[31] Canoso RT, Sise HS (1982). Chlorpromazine-induced lupus anticoagulant and associated immunologic abnormalities. Am J Hematol.

[32] Canoso RT, de Oliveira RM (1986). Characterization and antigenic specificity of chlorpromazine-induced antinuclear antibodies. J Lab Clin Med.

[33] Mordal J, Medhus S, Holm B, Morland J, Bramness JG (2013). Influence of drugs of abuse and alcohol upon patients admitted to acute psychiatric wards: physician’s assessment compared to blood drug concentrations. J Clin Psychopharmacol.

[34] Kohigashi M, Kitabayashi Y, Okamura A, Nakamura M, Hoshiyama A, Kunizawa M (2013). Relationship between patients’ quality of life and coercion in psychiatric acute wards. Psychiatry Res.

[35] Pruss H, Holtje M, Maier N, Gomez A, Buchert R, Harms L (2012). IgA NMDA receptor antibodies are markers of synaptic immunity in slow cognitive impairment. Neurology.

[36] Pruss H, Finke C, Holtje M, Hofmann J, Klingbeil C, Probst C (2012). *N*-methyl-d-aspartate receptor antibodies in herpes simplex encephalitis. Ann Neurol.

[37] Zerche M, Weissenborn K, Ott C, Dere E, Asif AR, Worthmann H (2015). Preexisting serum autoantibodies against the NMDAR Subunit NR1 modulate evolution of lesion size in acute ischemic stroke. Stroke.

[38] Hara M, Martinez-Hernandez E, Arino H, Armangue T, Spatola M, Petit-Pedrol M (2018). Clinical and pathogenic significance of IgG, IgA, and IgM antibodies against the NMDA receptor. Neurology.

