# Supplementary material for: The significance of anti-neuronal antibodies for acute psychiatric disorders: a retrospective case–controlled study
Source: BMC Neurosci. 2018 Nov 3;19:68. doi: 10.1186/s12868-018-0471-7 (PMC6215671; doi:10.1186/s12868-018-0471-7)
Supplement: Supplementary file 1 — Additional file 1.Search strategy for selection of variables: search strategy and citations reviewed during variable selection; Anti-neuronal antibody status in cases: antibody type, subtype and titer in all cases. [file 12868_2018_471_MOESM1_ESM.doc]

Supplementary file

**The significance of anti-neuronal antibodies for acute psychiatric disorders – a case-controlled study**

M. Schou, S.G. Sæther, O.K. Drange, K. Krane-Gartiser, S.K. Reitan, A.E. Vaaler, D. Kondziella

Search strategy for *Selection of variables in the study*

We searched Medline for relevant literature reporting on psychiatric symptoms in patients with disorders associated to NMDAR, GAD and CASPR2 antibodies. The search was performed the 14 October 2016. We used the following search strategy: (anti-GAD or "GAD antibodies") or (anti-CASPR2 or "CASPR2 antibodies") or (anti-VGKC or "VGKC antibodies") or ("NMDAR antibodies" or anti-NMDAR) or “anti-neuronal antibodies”. The initial search revealed 1315 individual citations. We did not consider studies with n<5, studies reporting on pediatric or adolescent patients (age < 18 years), or non-English literature. In papers with relevant titles, abstracts were evaluated. 40 individual citations were deemed eligible studies on the basis of their full text (references are found below). One author (SGS) extracted the relevant clinical characteristics (the variables in the present study), which was validated by a second author (OKD).

References for variable selection

1. Dalmau J, Lancaster E, Martinez-Hernandez E, Rosenfeld MR, Balice-Gordon R: Clinical experience and laboratory investigations in patients with anti-NMDAR encephalitis. *Lancet neurology* 2011, 10(1):63-74.

2. Chen X, Li JM, Liu F, Wang Q, Zhou D, Lai X: Anti-N-methyl-D-aspartate receptor encephalitis: a common cause of encephalitis in the intensive care unit. *Neurological sciences : official journal of the Italian Neurological Society and of the Italian Society of Clinical Neurophysiology* 2016.

3. Finke C, Kopp UA, Pruss H, Dalmau J, Wandinger KP, Ploner CJ: Cognitive deficits following anti-NMDA receptor encephalitis. *Journal of neurology, neurosurgery, and psychiatry* 2012, 83(2):195-198.

4. Gabilondo I, Saiz A, Galan L, Gonzalez V, Jadraque R, Sabater L *et al*: Analysis of relapses in anti-NMDAR encephalitis. *Neurology* 2011, 77(10):996-999.

5. Gable MS, Gavali S, Radner A, Tilley DH, Lee B, Dyner L *et al*: Anti-NMDA receptor encephalitis: report of ten cases and comparison with viral encephalitis. *European journal of clinical microbiology & infectious diseases : official publication of the European Society of Clinical Microbiology* 2009, 28(12):1421-1429.

6. Huang X, Fan C, Wu J, Ye J, Zhan S, Song H *et al*: Clinical analysis on anti-N-methyl-D-aspartate receptor encephalitis cases: Chinese experience. *Int J Clin Exp Med* 2015, 8(10):18927-18935.

7. Kamble N, Netravathi M, Saini J, Mahadevan A, Yadav R, Nalini A *et al*: Clinical and imaging characteristics of 16 patients with autoimmune neuronal synaptic encephalitis. *Neurology India* 2015, 63(5):687-696.

8. Kayser MS, Titulaer MJ, Gresa-Arribas N, Dalmau J: Frequency and characteristics of isolated psychiatric episodes in anti-N-methyl-d-aspartate receptor encephalitis. *JAMA neurology* 2013, 70(9):1133-1139.

9. Kruse JL, Jeffrey JK, Davis MC, Dearlove J, IsHak WW, Brooks JO, 3rd: Anti-N-methyl-D-aspartate receptor encephalitis: a targeted review of clinical presentation, diagnosis, and approaches to psychopharmacologic management. *Annals of clinical psychiatry : official journal of the American Academy of Clinical Psychiatrists* 2014, 26(2):111-119.

10. Kuppuswamy PS, Takala CR, Sola CL: Management of psychiatric symptoms in anti-NMDAR encephalitis: a case series, literature review and future directions. *General hospital psychiatry* 2014, 36(4):388-391.

11. Lejuste F, Thomas L, Picard G, Desestret V, Ducray F, Rogemond V *et al*: Neuroleptic intolerance in patients with anti-NMDAR encephalitis. *Neurology(R) neuroimmunology & neuroinflammation* 2016, 3(5):e280.

12. Leon-Caballero J, Pacchiarotti I, Murru A, Valenti M, Colom F, Benach B *et al*: Bipolar disorder and antibodies against the N-methyl-d-aspartate receptor: A gate to the involvement of autoimmunity in the pathophysiology of bipolar illness. *Neuroscience and biobehavioral reviews* 2015.

13. Leypoldt F, Armangue T, Dalmau J: Autoimmune encephalopathies. *Annals of the New York Academy of Sciences* 2015, 1338:94-114.

14. Lim JA, Lee ST, Jung KH, Kim S, Shin JW, Moon J *et al*: Anti-N-methyl-d-aspartate receptor encephalitis in Korea: clinical features, treatment, and outcome. *J Clin Neurol* 2014, 10(2):157-161.

15. Liu J, Wang D, Xiong Y, Liu B, Liu M: Anti-NMDAR Encephalitis of 11 Cases in China - Detailed Clinical, Laboratory and Imagiological Description. *European neurology* 2015, 74(1-2):73-78.

16. McKeon GL, Scott JG, Spooner DM, Ryan AE, Blum S, Gillis D *et al*: Cognitive and Social Functioning Deficits after Anti-N-Methyl-D-Aspartate Receptor Encephalitis: An Exploratory Case Series. *Journal of the International Neuropsychological Society : JINS* 2016, 22(8):828-838.

17. Maat P, de Graaff E, van Beveren NM, Hulsenboom E, Verdijk RM, Koorengevel K *et al*: Psychiatric phenomena as initial manifestation of encephalitis by anti-NMDAR antibodies. *Acta Neuropsychiatr* 2013, 25(3):128-136.

18. Pruss H, Holtje M, Maier N, Gomez A, Buchert R, Harms L *et al*: IgA NMDA receptor antibodies are markers of synaptic immunity in slow cognitive impairment. *Neurology* 2012, 78(22):1743-1753.

19. Sinmaz N, Amatoury M, Merheb V, Ramanathan S, Dale RC, Brilot F: Autoantibodies in movement and psychiatric disorders: updated concepts in detection methods, pathogenicity, and CNS entry. *Annals of the New York Academy of Sciences* 2015, 1351:22-38.

20. Titulaer MJ, McCracken L, Gabilondo I, Iizuka T, Kawachi I, Bataller L *et al*: Late-onset anti-NMDA receptor encephalitis. *Neurology* 2013, 81(12):1058-1063.

21. Tsutsui K, Kanbayashi T, Tanaka K, Boku S, Ito W, Tokunaga J *et al*: Anti-NMDA-receptor antibody detected in encephalitis, schizophrenia, and narcolepsy with psychotic features. *BMC psychiatry* 2012, 12:37.

22. Wang W, Li JM, Hu FY, Wang R, Hong Z, He L *et al*: Anti-NMDA receptor encephalitis: clinical characteristics, predictors of outcome and the knowledge gap in southwest China. *European journal of neurology : the official journal of the European Federation of Neurological Societies* 2016, 23(3):621-629.

23. Irani SR, Pettingill P, Kleopa KA, Schiza N, Waters P, Mazia C *et al*: Morvan syndrome: clinical and serological observations in 29 cases. *Annals of neurology* 2012, 72(2):241-255.

24. Joubert B, Saint-Martin M, Noraz N, Picard G, Rogemond V, Ducray F *et al*: Characterization of a Subtype of Autoimmune Encephalitis With Anti-Contactin-Associated Protein-like 2 Antibodies in the Cerebrospinal Fluid, Prominent Limbic Symptoms, and Seizures. *JAMA neurology* 2016, 73(9):1115-1124.

25. Lancaster E, Huijbers MG, Bar V, Boronat A, Wong A, Martinez-Hernandez E *et al*: Investigations of caspr2, an autoantigen of encephalitis and neuromyotonia. *Annals of neurology* 2011, 69(2):303-311.

26. Malter MP, Helmstaedter C, Urbach H, Vincent A, Bien CG: Antibodies to glutamic acid decarboxylase define a form of limbic encephalitis. *Annals of neurology* 2010, 67(4):470-478.

27. Pruss H, Lennox BR: Emerging psychiatric syndromes associated with antivoltage-gated potassium channel complex antibodies. *Journal of neurology, neurosurgery, and psychiatry* 2016, 87(11):1242-1247.

28. Sunwoo JS, Lee ST, Byun JI, Moon J, Shin JW, Jeong DE *et al*: Clinical manifestations of patients with CASPR2 antibodies. *Journal of neuroimmunology* 2015, 281:17-22.

29. van Sonderen A, Arino H, Petit-Pedrol M, Leypoldt F, Kortvelyessy P, Wandinger KP *et al*: The clinical spectrum of Caspr2 antibody-associated disease. *Neurology* 2016, 87(5):521-528.

30. Alexopoulos H, Dalakas MC: Immunology of stiff person syndrome and other GAD-associated neurological disorders. *Expert review of clinical immunology* 2013, 9(11):1043-1053.

31. Dayalu P, Teener JW: Stiff Person syndrome and other anti-GAD-associated neurologic disorders. *Seminars in neurology* 2012, 32(5):544-549.

32. Chang T, Alexopoulos H, McMenamin M, Carvajal-Gonzalez A, Alexander SK, Deacon R *et al*: Neuronal surface and glutamic acid decarboxylase autoantibodies in Nonparaneoplastic stiff person syndrome. *JAMA neurology* 2013, 70(9):1140-1149.

33. Dalakas MC, Fujii M, Li M, McElroy B: The clinical spectrum of anti-GAD antibody-positive patients with stiff-person syndrome. *Neurology* 2000, 55(10):1531-1535.

34. Fernandes M, Munhoz RP, Carrilho PE, Arruda WO, Lorenzoni PJ, Scola RH *et al*: Neurological disorders associated with glutamic acid decarboxylase antibodies: a Brazilian series. *Arquivos de neuro-psiquiatria* 2012, 70(9):657-661.

35. Mata S, Muscas GC, Cincotta M, Bartolozzi ML, Ambrosini S, Sorbi S: GAD antibodies associated neurological disorders: incidence and phenotype distribution among neurological inflammatory diseases. *Journal of neuroimmunology* 2010, 227(1-2):175-177.

36. Murinson BB: Stiff-person syndrome. *The neurologist* 2004, 10(3):131-137.

37. Murinson BB, Vincent A: Stiff-person syndrome: autoimmunity and the central nervous system. *CNS spectrums* 2001, 6(5):427-433.

38. Sarva H, Deik A, Ullah A, Severt WL: Clinical Spectrum of Stiff Person Syndrome: A Review of Recent Reports. *Tremor Other Hyperkinet Mov (N Y)* 2016, 6:340.

39. Takagi M, Ishigaki Y, Uno K, Sawada S, Imai J, Kaneko K *et al*: Cognitive dysfunction associated with anti-glutamic acid decarboxylase autoimmunity: a case-control study. *BMC neurology* 2013, 13:76.

40. Tohid H: Anti-glutamic acid decarboxylase antibody positive neurological syndromes. *Neurosciences (Riyadh)* 2016, 21(3):215-222.

Anti-neuronal antibody and endpoint titer in cases

| Case | NMDA (Ig subtype and titer) | CASPR2 (Ig subtype and titer) | GAD65 (Ig subtype and titer) |
| --- | --- | --- | --- |
| 1 | M, 1:100 |  |  |
| 2 | G, 1:32 |  |  |
| 3 | G, 1:10 |  |  |
| 4 | A, 1:10 |  |  |
| 5 | A, 1:10 |  |  |
| 6 | A, 1:32 |  |  |
| 7 | M, 1:10 |  | G, 1:10 |
| 8 | A, 1:32 |  |  |
| 9 | A, 1:32 |  |  |
| 10 | A, 1:10 |  | G, 1:10 |
| 11 | M, 1:32 |  |  |
| 12 | M, 1:100 |  |  |
| 13 | M, 1:100 |  |  |
| 14 | M, 1:10 |  |  |
| 15 | A, 1:10 |  |  |
| 16 | A, 1:10 |  |  |
| 17 | M, 1:320 |  | G, 1:10 |
| 18 | A, 1:10 |  |  |
| 19 | M, 1:100, A, 1:10 |  |  |
| 20 | G, 1:100 |  |  |
| 21 | M, 1:1000, A, 1:1000 |  |  |
| 22 |  | G, 1:10 |  |
| 23 |  | M, 1:10 |  |
| 24 |  | M, 1:10 |  |
| 25 |  | A, 1:100 |  |
| 26 |  | M, 1:32 |  |
| 27 |  | G, 1:100 |  |
| 28 |  | A, 1:10 |  |
| 29 |  | G, 1:32 |  |
| 30 |  | M, 1:32 |  |
| 31 |  | M, 1:10 |  |
| 32 |  | M, 1:10 |  |
| 33 |  | G, 1:32 |  |
| 34 |  | G, 1:10 |  |
| 35 |  | G, 1:10 |  |
| 36 |  |  | G, 1:320 |
| 37 |  |  | A, 1:100 |
| 38 |  |  | G, 1:100 |
| 39 |  |  | G, 1:10 |
| 40 |  |  | G, 1:10 |
| 41 |  |  | G, 1:100 |
